# Current Status of Human Papillomavirus-Related Head and Neck Cancer: From Viral Genome to Patient Care

**DOI:** 10.1007/s12250-021-00413-8

**Published:** 2021-06-21

**Authors:** Haoru Dong, Xinhua Shu, Qiang Xu, Chen Zhu, Andreas M. Kaufmann, Zhi-Ming Zheng, Andreas E. Albers, Xu Qian

**Affiliations:** 1 Department of Clinical Laboratory, Cancer Hospital of the University of Chinese Academy of Sciences (Zhejiang Cancer Hospital), Institute of Basic Medicine and Cancer (IBMC), Chinese Academy of Sciences, Hangzhou, 310022 China; 2 Cancer Hospital of the University of Chinese Academy of Sciences (Zhejiang Cancer Hospital), Institute of Basic Medicine and Cancer (IBMC), Chinese Academy of Sciences, Hangzhou, 310022 China; 3 Department of Cancer Prevention, Cancer Hospital University of Chinese Academy of Sciences (Zhejiang Cancer Hospital), Institute of Basic Medicine and Cancer (IBMC), Chinese Academy of Sciences, Hangzhou, 310022 China; 4 Clinic for Gynecology, Berlin Institute of Health, Charité-Universitätsmedizin Berlin, Freie Universität Berlin, Humboldt-Universität Zu Berlin, Berlin, 12203 Germany; 5 Tumor Virus RNA Biology Section, HIV Dynamics and Replication Program, National Cancer Institute, National Institutes of Health, Frederick, MD 21702 USA; 6 Department of Otolaryngology, Head and Neck Surgery, Berlin Institute of Health, Charité-Universitätsmedizin Berlin, Freie Universität Berlin, Humboldt-Universität Zu Berlin, Berlin, 13353 Germany

**Keywords:** Human papillomavirus (HPV) integration; Molecular diagnostics, Oropharyngeal cancer, De-Escalation treatment, Immunotherapy

## Abstract

Human papillomavirus (HPV) infection identified as a definitive human carcinogen is increasingly being recognized for its role in carcinogenesis of human cancers. Up to 38%–80% of head and neck squamous cell carcinoma (HNSCC) in oropharyngeal location (OPSCC) and nearly all cervical cancers contain the HPV genome which is implicated in causing cancer through its oncoproteins E6 and E7. Given by the biologically distinct HPV-related OPSCC and a more favorable prognosis compared to HPV-negative tumors, clinical trials on de-escalation treatment strategies for these patients have been studied. It is therefore raised the questions for the patient stratification if treatment de-escalation is feasible. Moreover, understanding the crosstalk of HPV-mediated malignancy and immunity with clinical insights from the proportional response rate to immune checkpoint blockade treatments in patients with HNSCC is of importance to substantially improve the treatment efficacy. This review discusses the biology of HPV-related HNSCC as well as successful clinically findings with promising candidates in the pipeline for future directions. With the advent of various sequencing technologies, further biomolecules associated with HPV-related HNSCC progression are currently being identified to be used as potential biomarkers or targets for clinical decisions throughout the continuum of cancer care.

## Introduction

High-risk human papillomavirus (HR-HPV) infection leads to the development of human cancers in a variety of anatomical squamous tissue sites, including the head and neck regions. The incidence of HPV-related head and neck squamous cell carcinoma (HNSCC), especially oropharyngeal squamous cell carcinoma (OPSCC), has increased in the United States and Europe (Chaturvedi *et al.* 2013; Tinhofer *et al.* 2015; Wittekindt *et al.* 2019; Zamani *et al.* 2020). In some regions, HPV-related OPSCC accounts for 38%–80% of OPSCCs and 30% of HNSCCs (Boscolo-Rizzo *et al.* 2013; Chaturvedi *et al.* 2013; de Martel *et al.* 2020; Leemans *et al.* 2018) (Table 1). In particular, patients with HPV-related OPSCC have a better prognosis than those with HPV-negative OPSCC (Leemans *et al.* 2018). The proportion of comparatively younger patients is high among patients with HPV-related OPSCC (Leemans *et al.* 2018). Moreover, HPV-related OPSCCs are more sensitive to chemoradiotherapy and immune checkpoint blockade (ICB) treatments than HPV-unrelated tumors (Leemans *et al.* 2018). With the accumulated understanding of the new entity of HPV-related OPSCC, a new staging system in the 8th edition of the American Joint Committee on Cancer (AJCC) has been established toward a deintensified treatment protocol to reduce long-term associated morbidity for patients with HPV-related OPSCC. However, recent phase III clinical trials (RTOG 1016 and De-ESCALaTE) have shown that a number of patients with HPV-related OPSCC who received deintensified treatment had an inferior outcome compared to those who received standard care (Gillison *et al.* 2019b; Mehanna *et al.* 2019). Moreover, patients with HPV-related HNSCC at multiple sites, defined as one HPV-positive primary OPSCC and a second primary of any head and neck site, demonstrated distinct characteristics (i.e., a lower T and N stage) compared to patients with one primary tumor (Joseph *et al.* 2013; Strober *et al.* 2020). These observations lead to a further discussion on the feasibility of the current design of the de-escalation treatment protocol in clinical trials and highlight the necessity to refine the stratification strategy for patients with HPV-related HNSCC, in particular for OPSCC (Ventz *et al.* 2019).

**Table 1:** Human papillomavirus (HPV) status in relation to oropharyngeal squamous cell carcinoma (OPSCC).

	Time interval of collected data	Samples	Men/women	Median Age	HPV genotypes	Detection method	HPV + rate	References
China								
	2012–2018	49	29/20	53	16, 18, 52	HPV 16/18 RNA ISH	28.6%	Yang *et al.* (2020)
	2007–2019	152	127/25	NA	11, 16, 18, 33, 53, 58	HPV genotyping	65.1%	Xu *et al.* (2020c)
	2014–2019	257	221/36	60	NA	HPV Genotyping	18.3%	Xu *et al.* (2020b)
	1999–2013	300	273/27	54	16, 33, 35, 56, 58, 68	HPV Genotyping and/or p16 IHC	25.0%	Chen *et al.* (2020)
USA								
	2010–2014	1168	926/242	61	NA	HPV 16/18 DNA ISH and/or p16 IHC	52.8%	White *et al.* (2020)
	2010–2016	45,940	38,0381/7902	60	16, 18, 31, 33, 35, 36, 45, 51, 52, 56, 58, 59, 68, 26, 53, 66, 67, 69, 70, 73, 82, 85	HPV 16/18 DNA ISH and/or p16 IHC	67.5%	Rotsides *et al.* (2020)
	1996–2013	115	101/14	56	NA	p16 IHC	90.4%	Altenhofen *et al.* (2020)
	NA	381	332/49	NA	NA	HPV RNA	79.8%	Liu *et al.* (2020)
	2002–2013	611	517/94	NA	NA	HPV ISH and p16 IHC	89.0%	Elhalawani *et al.* (2020)
	2007–2018	88	69/19	73	16, 18, 33, 35	PCR amplification of HPV gene loci or p16 IHC	70.5%	Dickstein *et al.* (2020)
UK								
	2010–2016	273	207/66	59	NA	p16 IHC	73.3%	De Felice *et al.* (2020)
Canada								
	2005–2017	2039	1668/371	NA	NA	p16-IHC	48.7%	Huang *et al.* (2020)
	1997–2015	372	0/372^a^	NA	NA	p16-IHC	56.8%	Gazzaz *et al.* (2019)
	1998–2004	525	381/144	NA	NA	p16-IHC, HPV DNA ISH	73.5%	Hall *et al.* (2019)
Australia								
	2016–2017	650	284/366	52	16	HPV16 DNA	1.8%	Tang *et al.* (2020)
	2018–2019	910	315/595	37	13, 16, 18, 32	HPV DNA	35.3%	Jamieson *et al.* (2020)
Australia and New Zealand								
	NA	189	NA	NA	16, 18	P16 IHC and HPVRNA ISH	88.1%	Young *et al.* (2020a, 2020b)
Netherlands								
	2009–2016	216	143/73	NA	NA	p16 IHC and HPV DNA	31.9%	Chargi *et al.* (2020)
	1995–2015	168	135/33	NA	NA	p16 IHC and/or HPV DNA	50.0%	Molony *et al.* (2020)
Brazil								
	1999–2010	346	308/38	55	16	HPV16 and p16 IHC	6.1%	Buexm *et al.* (2020)
	2017–2019	91	78/13	61	NA	p16 IHC	20.9%	Girardi *et al.* (2020)
	1984–2014	215	190/25	56	16, 18, 31, 33, 35, 39, 45, 51, 52, 56, 58, 59, 68, 73, 82	HPV DNA detection and/or p16 IHC	59.1%	De Cicco *et al.* (2020)
Germany								
	2000–2017	102	82/20	57.5	16, 18, 33, 59	HPV genotyping and p16 IHC	40.2%	Weiss *et al.* (2020)
	2000–2011	141	103/38	NA	16	HPV DNA and p16 IHC	34.0%	Huebbers *et al.* (2019)
	2000–2014	323	245/78	58.87	NA	HPV DNA and p16 IHC	19.8%	Grønhøj *et al.* (2019a)
	2000–2017	730	NA	NA	NA	HPV DNA and p16 IHC	27.1%	Wittekindt *et al.* (2019)
	2007–2016	92	77/15	NA	16, 18	HPV Genotyping and p16 IHC	71.4%	Freitag *et al.* (2020)
Denmark								
	2000–2017	2169	1564/605	58	16, 18, 33, 25, 59, 26, 31, 45, 56, 11, 58	HPV DNA detection and p16 IHC	55.0%	Zamani *et al.* (2020)
	2000–2014	417	0/417	61.2	NA	HPV DNA and p16 IHC	48.7%	Christensen *et al.* (2019)
	2000–2014	993	720/273	59.50	NA	HPV DNA and p16 IHC	56.9%	Grønhøj *et al.* (2019a)
	2000–2014	1499	NA	NA	NA	HPV DNA and p16 IHC	55.0%	Grønhøj *et al.* (2019b)
	2000–2014	1243	903/340	60.2	NA	HPV DNA and p16 IHC	63.4%	Rasmussen *et al.* (2019)
Korea								
	NA	60	50/10	59	NA	HPV DNA detection and p16 IHC	80.0%	Suh *et al.* (2020)
	2004–2013	113	101/12	NA	NA	HPV Genotyping and p16 IHC	69.9%	Kwon *et al.* (2020)
Austria								
	2014–2019	62	48/14	NA	16, 18, 33, 40, 62, 68	HPV Genotyping or p16 IHC	100%	Kofler *et al.* (2020)
South Glasgow								
	2010–2017	272	NA	NA	16. 18. 33. 39. 58	HPV Genotyping	44.0%	Zubair *et al.* (2020)
Finland								
	2000–2016	157	110/47	59.5	NA	p16 IHC	9.8%	Sievert *et al.* (2020)
Spain								
	2017–2019	54	43/11	62	NA	p16 IHC and HPV DNA	18.5%	Viros Porcuna *et al.* (2020)
Cameroon								
	2014–2015	101	31/70	42	32, 68, 82	p16 IHC and HPV RNA ISH	5.0%	Rettig *et al.* (2019)
Sweden								
	2000–2018	195	123/72	67	NA	HPV DNA and p16 IHC	15.9%	Hammarstedt *et al.* (2020)
Croatia								
	2002–2015	99	82/17	60	NA	HPV DNA and HPV E6 mRNA	40.4%	Božinović *et al.* (2019)
Thailand								
	2010–2016	110	95/15	59	16, 18, 26, 31, 33, 35, 39, 45, 51, 52, 53, 56, 58, 59, 66, 68, 70, 73, 82	p16 IHC and HPV DNA ISH	14.5%	Nopmaneepaisarn *et al.* (2019)
Italy								
	2010–2017	59	43/16	66	NA	HPV DNA	41.2%	Ravanelli *et al.* (2020)

Studies included in this table meet the following criteria: OPSCC patients; HPV DNA or RNA positive or p16 positive; studies published between January 2019 and October 2020

^a^This study included women with OPSCC only

IHC: immunohistochemical staining; ISH: in situ hybridization

HPV-driven oncogenic processes are characterized by HPV oncoproteins E6 and E7, which induce p53 and retinoblastoma (Rb) degradation, consequently leading to a deregulation of the cell cycle and an inhibition of apoptosis (Wittekindt *et al.* 2018). A plethora of data have been recently accumulated for different incidences of gene mutations and chromosomal aberrations between HPV-related and HPV-unrelated HNSCCs (Pickering *et al.* 2013; The Cancer Genome Atlas Network 2015). For example, TP53 mutations are found in approximately 60%–70% of HNSCCs, and different gain-of-function p53 mutants are related to oncogenesis, especially in HPV-unrelated HNSCC (Zhou *et al.* 2016). We recently developed a prognostic scoring system including five covariates (age, pT, pN, perineural invasion, and EAp53 score) for HPV-independent HNSCC patients via The Cancer Genome Atlas (TCGA)-based tumor genomic analysis (Qian *et al.* 2019). In addition to TP53 mutation, the exclusivity of CDKN2A and TERT driver mutations has also been identified in HPV-related HNSCC (Zapatka *et al.* 2020). Recurrent deletions and truncating mutations of TNF receptor-associated factor 3 (TRAF3), which is involved in innate and acquired antiviral immune responses, were found to be associated with HPV-related HNSCC (The Cancer Genome Atlas Network 2015). Furthermore, recent omics studies on HPV virus-host protein interactions have identified several potential and multiple oncogenesis pathways that can be promoted by HPV interactions, similar to recurrent mutations in cancer (Eckhardt *et al.* 2018). Moreover, an increase in mutations related to a higher expression of apolipoprotein B mRNA-editing catalytic polypeptide (APOBEC) was found in HPV-related HNSCC compared to HPV-negative tumors (Zapatka *et al.* 2020). Notably, it is hypothesized that a pathogenetic process is needed for the development of HPV-related cancers (Cui *et al.* 2019). A recent study demonstrated that genotypes of KRAS mutations and a loss of PTEN with both HPV E6/E7 dependence and independence lead to precipitous cervical cancer development, while HPV E6/E7 alone leads to only carcinoma in situ in a mouse model (Böttinger *et al.* 2020). A comprehensive understanding of how these mutations are involved in the carcinogenesis of HPV-related cancers remains to be established.

Another important consideration is antiviral defenses and host–pathogen interactions that help us understand HPV-induced immune evasions in HPV-related cancers (Zhou *et al.* 2019). E6 and/or E7 seropositivity has been found in the majority of HPV DNA-positive HNSCC patients and is associated with longer recurrence-free survival (Smith *et al.* 2010; Lang Kuhs *et al.* 2017). A genome-wide association study on the association between OPSCC and human leukocyte antigen (HLA) loci demonstrated that the class II haplotype DRB1*1301-DQA1*0103-DQB1*0603 was associated with a strongly reduced risk of HPV-related OPSCC compared to HPV-negative tumors (Lesseur *et al.* 2016).

However, the down-regulation of antigen-processing machinery components against HPV oncoproteins (i.e., HPV16 E7 or E5) has been reported in cervical cancer and HNSCC (Albers *et al.* 2005). HPV16 E7 can also suppress stimulator interferon gene (STING) complex-induced type I interferon (IFN-I) activation, by which effector T cell expansion is limited (Luo *et al.* 2020). Moreover, higher membranous PD-L1 expression at the tonsils and high levels of PD-1 expression within the majority of CD8 + tumor-infiltrating leukocytes (TILs) indicate adaptive immune resistance in HPV-related HNSCC (Lyford-Pike *et al.* 2013). In addition, the immune features within the tumor microenvironment (TME) of HPV-related HNSCC can be differentiated from HPV-negative tumors (Cillo *et al.* 2020). HPV-related OPSCC patients with HPV16-specific type-I T cells and type-I-oriented TME have a better prognosis than patients lacking HPV immunity (Welters *et al.* 2018). Enriched germinal center B cells in TILs of HPV-related HNSCC indicate their role through germinal center reactions during virus-driven progression (Cillo *et al.* 2020). B cells in nongerminal center states are more prominent in HPV-negative tumors (Cillo *et al.* 2020). Recently, HPV-specific B cell responses, including antigen-specific activated and germinal center B cells and plasma cells, were identified in the TME of samples from HPV-related HNSCC (Wieland *et al.* 2020). These findings demonstrate more heterogeneous immunity in HPV-driven tumors, which has the potential to refine the risk group.

A fundamental understanding of the heterogeneity, plasticity and cellular mechanisms of HPV-related HNSCC biology offers an opportunity to uncover therapeutic windows and to separate the small subset of patients with HPV-related cancer at high risk, from whom a de-escalation approach would be appropriate. Here, we review the current knowledge regarding HPV-related HNSCC and the challenges of targeting these cancers. We also discuss potential applications for biomarker-based stratification strategies, which are due to spatial and temporal varieties of conditions, including the broader TME and the underlying pathways of antitumor response and tumor resistance.

## The Epidemiology of HPV Oral Infections and HNSCC

Worldwide, more than 830,000 cases of HNSCC are diagnosed each year, with approximately 430,000 deaths (Bray *et al.* 2018). The incidence of HPV-related HNSCC is increasing, i.e., by 2.5% per year for OPSCC in the United States (Mifsud *et al.* 2017; Mourad *et al.* 2017). In China, the estimated age-standardized incidence rate of HNSCC was 2.7 per 100,000 and 2.22/100,000 person-years by the standard population of China in 2000 (ASRIC and ASRMC) for OPSCC (Liu *et al.* 2018). As seen in recent studies in China, the rates of HR-HPV infection in HNSCC are 7.5% in a case control study and 26.4% in southern Chinese population (Chor *et al.* 2016; Ni *et al.* 2019). HPV-related HNSCCs have been found in the oral cavity, oropharynx, pharynx, larynx and salivary glands, with the highest prevalence for OPSCCs (Qian *et al.* 2016; Wiegand *et al.* 2018). Table 1 illustrates recent reports of HPV-related OPSCCs in different regions. Squamous cell carcinoma of unknown primary in the head and neck (SCCUPHN) present with neck lymph node metastasis with no evidence of a primary tumor accounts for 4%–5% of HNSCCs (Ren *et al.* 2019; Schroeder *et al.* 2020). The prevalence of HPV positivity in SCCUPHN was 49% as reviewed by a recent meta-analysis (Ren *et al.* 2019). In a prospective study, SCCUPHN, defined as the metastasis of SCC to a neck node, was likely to be HPV-driven OPSCC because of the similarities in risk factor profile and survival with HPV-driven OPSCC (Schroeder *et al.* 2020). Additionally, the rate of a secondary primary tumor in HNSCC with unknown HPV status is reported to be ~ 14%, and the highest is 36% (Chuang *et al.* 2008). Patients who develop multiple primary tumors of HPV-related HNSCC (one primary HPV-related OPSCC with a second primary tumor of any head and neck site) were recently reported at a rate of 0.95%–2.64% depending on different datasets (Strober *et al.* 2020). Notably, those patients tend to be younger and have no neck adenopathy compared to patients with one primary HPV-related tumor (Strober *et al.* 2020).

The prevalence of HPV-related OPSCC varies in different age groups. In earlier reports, patients with HPV-related OPSCC tended to be demographically younger. However, an increase in the proportion of patients of older age over time leads to the median age being older (Smith *et al.* 2004; Fakhry *et al.* 2020). Among recent studies from different regions, the median age varies from 37 to 73 years with the different time intervals of collected data (Table 1).

**Fig. 1:**
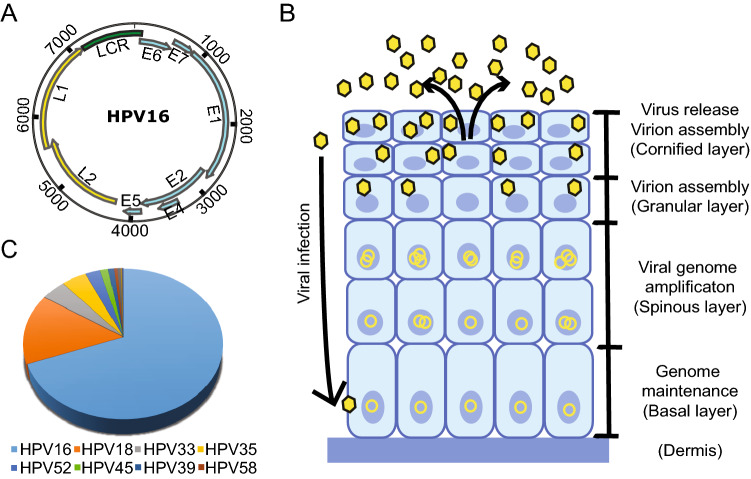
**A** The HPV genomic structure (use HPV 16 as an example). The ORFs encoding the early (blue) and late (yellow) genes are marked. **B** The life cycle of HPV starts with viral infection at the basal cells through trauma and the viral genomes are maintained at low copy number level in undifferentiated cell. Upon cell differentiation, the viral genomes become amplified and virion assembly ensues, resulting in the release of viruses in the cornified layer. **C** The contribution of different high-risk HPV genotypes to head and neck squamous carcinoma (Tumban 2019).

To date, HR-HPV genotypes 16, 18, 31, 33, 35, 39, 45, 51, 52, 56, 58, 59 are categorized as Group 1 carcinogens, 68 as Group 2A and 26, 30, 34, 53, 66, 67, 69, 70, 73, 82, 85, 97 as Group 2B carcinogen (Bouvard *et al.* 2009). HPV infections with HR-subtypes (HPV 16, 18, 26, 30, 31, 33, 35, 39, 45, 51, 52, 53, 56, 58, 59, 66, 67, 68 and 69) have been identified in HNSCC (Fig. 1, Table 1) (Castellsagué *et al.* 2016; Tumban 2019). A recent analysis of 3,680 samples from 29 countries shows that HPV 16, 33, 35 and 18 are responsible for the majority of HPV-related OPSCCs (Castellsagué *et al.* 2016). Overall, HPV 16 is predominant and accounts for 90%–97% of HPV-related OPSCCs (Gillison *et al.* 2015). Human immunodeficiency virus (HIV)-infected individuals have a risk of developing HPV-HIV coinfection, and the incidence of age-standardized HPV-related HNSCC increased from 6.8 to 11.4 per 100,000 person-years from 1996 to 2009 in North America (Beachler *et al.* 2014; Dsouza *et al.* 2014).

## HPV Genome and Expression

The genetic information of HPVs is carried by their circular, double-stranded DNA genome, which has a size of approximately 8,000 base pairs (Zheng and Baker 2006). According to the genome data compiled in the Papillomavirus Episteme database (https://pave.niaid.nih.gov/), there are more than 225 distinct genotypes of HPVs, which are classified according to the DNA sequence of the *L1* gene. To be recognized as a new genotype, the *L1* region sequence has to be more than 10% different from its closest member. Despite the sequence variation, the HPV genome structure is similar for all genotypes and is organized into three regions (Fig. 1). The early gene region contains several overlapping open reading frames (ORFs) denoted E1, E2, E4, E5, E6, E7 and E8 (E5 and E8 not present in all genotypes) that code for nonstructural and regulatory proteins involved in various processes of the virus life cycle, such as viral replication and transactivation of gene expression. Currently, 12–15 genotypes of HPVs are considered oncogenic for certain types of cancers, with the early proteins E6 and E7 playing key roles in oncogenesis and thus are two multifunctional viral oncoproteins (Zheng and Wang 2011; Roman and Munger 2013; Vande Pol and Klingelhutz 2013; Wang *et al.* 2014). The late gene region encodes structural proteins L1 and L2, the major and minor capsid proteins. In addition, a noncoding region (NCR) or upstream regulation region (URR) between the late and early regions contains a viral replication Ori (Wang *et al.* 2017) and binding sites for various viral and host transcription and regulation factors, such as viral E1 and E2, the functions of which are essential in viral DNA replication and amplification.

The transcription of viral genes and viral genome replication are tightly regulated throughout the life cycle of papillomaviruses in a host cell differentiation manner. The virus infects basal layer cells through epithelial layer trauma. In undifferentiated basal cells, the expression of most viral early proteins from viral early promoter-derived RNA transcripts is maintained at low levels to avoid triggering immune responses. As cell differentiation occurs, viral DNA replication and activation of the viral late promoter lead to the expression of viral late genes and the production of infectious virus particles in the granular and cornified layers of the epithelium (Fig. 1B). Although epigenetic modifications, including chromatin remodeling and DNA methylation on both viral and host genomes, play important roles in the control of viral gene expression, with numerous studies being explored for their function at various stages of the viral life cycle, the posttranscriptional regulation, such as RNA splicing, polyadenylation, stability, export and translation, is essential for the expression of each viral protein. Our understanding of these regulations remains minimal. In high-risk HPV16 and HPV18, viral E6 and E7 are expressed as a single bicistronic E6/E7 RNA undergoing extensive RNA splicing (Tang *et al.* 2006; Wang *et al.* 2011; Ajiro *et al.* 2016). While viral E6 is expressed from the unspliced E6 RNA coding region, viral E7 protein can be expressed only from spliced E6*I RNA (Zheng *et al.* 2004; Tang *et al.* 2006; Ajiro *et al.* 2012; Brant *et al.* 2019). To date, we know very little how viral E1 and E2 are expressed in the context of the entire viral genome during high-risk HPV infection. Papillomaviral E4 is encoded from the viral early region, but it is a viral late protein translated from a viral late transcript derived from a late promoter residing in the E7 coding region (Wang *et al.* 2011; Xue *et al.* 2017). By alternative splicing, this late transcript also translates viral L2 and L1 for viral capsid formation (Zheng and Baker 2006). Viral L1 and L2 form the basis for HPV vaccines in preventing HPV infection and the development of HPV-induced cancers (Zhou *et al.* 1991; Kirnbauer *et al.* 1992; Schiller *et al.* 2012). Further elucidation of the molecular mechanisms that control viral DNA replication, transcription and posttranscriptional regulation may provide novel targets for combating HPV and treating HPV-associated cancers. For more detailed discussion on epigenetic regulation of HPV genome transcription, please refer to a recent review by Burley et al*.* (Burley *et al.* 2020).

## HPV Integration and HNSCC

The integration of HR-HPV DNA into the host genome has been considered an important biological step in the development of carcinogenesis in invasive cervical cancer and HNSCC (Mesri *et al.* 2014; Zapatka *et al.* 2020). Initial studies demonstrated that transcriptionally active integrated and/or episomal viral DNA in HNSCC cell lines was independent of viral copy number and integration sites (Akagi *et al.* 2014; Olthof *et al.* 2015). HPV integration can lead to host genomic instability, such as deletions, inversions, and chromosomal translocations (Akagi *et al.* 2014). A number of viral integration sites in the host genome were found in intergenic regions as well as cancer-associated genes such as TP63, ETS2, RUNX1, FOXA1 and ERBB2 (Olthof *et al.* 2014, 2015; Walline *et al.* 2016; Koneva *et al.* 2018). Moreover, viral integration into cellular genes was commonly identified in recurrent HPV16-positive OPSCC patients, and these cellular genes are related to cancer-associated signaling pathways or mechanisms (Walline *et al.* 2016). Integrated viral DNA copies could be in tandem. Viral DNA integration through the disruption of the viral E2 region leads to increased transcription of viral E6 and E7. Tumors with HPV DNA integration differ from HPV integration-negative tumors by different patterns of DNA methylation and gene expression profiles (Parfenov *et al.* 2014). Recently, Zapatka *et al.* found that HPV 16 and HPV 18 integration events in cervical cancer and HNSCC were associated with local variations and genomic rearrangements based on the Pan-Cancer Analysis of Whole Genomes Consortium (Zapatka *et al.* 2020).

HPV integration inducing genome instability is hypothesized to be a secondary genetic event in the carcinogenesis of HPV-associated HNSCC. HPV infection is associated with increased expression of the *APOBEC* genes *APOBEC3A* and *APOBEC3B* but exclusively with known driver genes such as *TP53*, *CDKN2A* and *TERT* (Kondo *et al.* 2017; Zapatka *et al.* 2020). These findings suggest a possible role of APOBECs in HPV-induced carcinogenesis, i.e., the activity of APOBECs as C-to-U RNA editing enzymes contributes to alterations in host genome expression, and APOBEC3A increases tumorigenesis *in vivo* (Burns *et al.* 2013; Wallace and Münger 2018; Law *et al.* 2020). In addition, as part of the immune defense system, APOBEC3A can sensitize cancer cells to cisplatin treatment by activating base excision repair and mediating the repair of cisplatin interstrand crosslinks (Conner *et al.* 2020). These results suggest a role of impaired antiviral defense in driving the carcinogenesis of HPV-related HNSCC. HPV16 insertions also lead to the amplification of the PIM1 serine/threonine kinase gene in HNSCC cell lines (Broutian *et al.* 2020). The inhibition of PIM family kinases successfully decreased cell proliferation *in vitro* and *in vivo* in an HNSCC model (Broutian *et al.* 2020).

Notably, viral integration can be found in both tumors that respond to treatment and recurrent tumors with more complex integration patterns in host genes (Walline *et al.* 2016). By analyzing viral-host fusion transcripts, Koneva *et al.* showed that the HPV-positive but HPV integration-negative subgroup had better survival than the HPV integration-positive subgroup and HPV-unrelated HNSCC (Koneva *et al.* 2018). Moreover, HPV-positive but HPV integration-negative tumors had enhanced tumor infiltrates of immune cells and upregulated immune-related genes. Consistently, another study indicated that HPV-related HNSCC can be subdivided into an immune cell enrichment phenotype and a phenotype with higher proliferation (Koneva *et al.* 2018). Thus, the enhanced immune profile in patients with HPV-positive but HPV integration-negative tumors may be attributed to better survival for these patients. However, potential mechanisms for HPV integration-induced oncogenesis of HNSCC remain elusive.

## Genetic and Epigenetic Alterations

Recent landmark sequencing studies have demonstrated gene expression profiles and somatic mutations such as *TP53*, *CDKN2A*, *PTEN*, *PIK3CA*, *EGFR*, *HRAS*, *FBXW7* and *NOTCH1* in diverse anatomical sites of HNSCC (Agrawal *et al.* 2011; Stransky *et al.* 2011; Pickering *et al.* 2013; The Cancer Genome Atlas Network 2015). Importantly, diversity in the number of mutations and gene profiles was seen in patients with a history of tobacco use and between HPV-related and HPV-unrelated tumors. The mutation rate of HPV-related tumors was almost half that of HPV-unrelated tumors (Stransky *et al.* 2011). Thus, two etiologies may result in the alteration of oncogenes and tumor suppressor genes that have tumorigenic effects involved in multistep biological processes.

HPV-related HNSCC harbors mutations in the oncogene *PIK3CA* encoding PI3K catalytic p110 subunit alpha, a loss of TRAF3 and the amplification of E2F1 (The Cancer Genome Atlas Network 2015). A recent comprehensive analysis on oral squamous cell carcinoma (OSCC) identified secondary genetic alterations, including *PIK3CA*, *ZNF750* and *EP300* as candidate cancer driver genes (Gillison *et al.* 2019a). *APOBEC* cytosine deaminase editing was associated with genomic mutation burden in HPV-related OSCC (Gillison *et al.* 2019a). *APOBEC*-mediated cytosine deamination leading to *PIK3CA* mutations is involved in the tumorigenesis of HPV-driven tumors (Henderson *et al.* 2014; Gillison *et al.* 2019a). As we discussed above, virus-host interactions, as seen by the interaction between HPV integration with *APOBEC* and others, may shape genomic alterations and facilitate tumorigenesis.

Notably, *PIK3CA* mutations (2.6% to 19%) lead to the activation of the PI3K-AKT-mTOR1 signaling pathway necessary for the viral life cycle (Lui *et al.* 2013; Surviladze *et al.* 2013). HPV oncoproteins E6 and E7 also increase PI3K-AKT-mTOR signaling (Pim *et al.* 2005; Contreras-Paredes *et al.* 2009). Moreover, both HPV-related and HPV-unrelated HNSCCs harbor *PIK3CA* mutations, and higher expression of PIK3CA in primary tumors is associated with tumor recurrence and chemo- and radioresistance (García-Escudero *et al.* 2018; Marquard and Jücker 2020). Thus, inhibitors targeting the PI3K-AKT-mTOR pathway have been developed for cancer therapies (Marquard and Jücker 2020). However, the clinical response rates remain modest in these studies and warrant further investigation (Marquard and Jücker 2020). Studies in patient-derived xenograft (PDX) models demonstrate that *EGFR*, *AKT1* and *CSMD1* copy number aberrations are related to the effect of PI3-kinase inhibition regardless of the status of *PIK3CA* mutation (Ruicci *et al.* 2019a). The knockdown of the TAM family receptor tyrosine kinases TYRO3 and AXL and the inhibition of MAPK signaling can resensitize resistance induced by alpelisib, a PI3K inhibitor (Ruicci *et al.* 2020). Adaptive resistance to PI3K inhibition is also seen in mTORC2-mediated Akt reactivation following PI3K inhibition. The knockdown of *RICTOR*, a subunit of mTORC2, can sensitize HNSCC cells to PI3K inhibition *in vitro* (Ruicci *et al.* 2019b).

TRAF3 functions as a tumor suppressor negatively to regulate NF-κB pathway activation in HPV-related HNSCC (Zhang *et al.* 2018). TRAF3 is involved in the innate and acquired antiviral immune responses (The Cancer Genome Atlas Network 2015). The re-expression of TRAF3 can enhance TP53 and RB tumor suppressor proteins and decrease HPV E6 oncoprotein in HPV + HNSCC cell lines (Zhang *et al.* 2018). Thus, regulating TRAF3 and aberrantly activating the alternative NF-kB pathway warrant further investigation as therapeutic targets in cancer treatment.

Another interesting aspect of HPV-induced HNSCC oncogenesis is epigenetic alterations, including DNA methylation and histone modifications (Kostareli *et al.* 2013; Hatano *et al.* 2017; Papillon-Cavanagh *et al.* 2017; Guo *et al.* 2020; Mac and Moody 2020). For example, higher DNA methylation levels are more common in HPV-related OPSCC than in HPV-unrelated tumors and normal tissues (Ren *et al.* 2018). Furthermore, candidates for DNA differentially methylated regions (DMRs) can discriminate HPV-related OPSCC from normal controls with good receiver operating characteristic (ROC) performances (Ren *et al.* 2018). Moreover, these changes are involved in different stages of the HPV life cycle in HPV-related OPSCC and other HPV-related malignancies (Boscolo-Rizzo *et al.* 2017; Mac and Moody 2020). HPV oncoproteins E6 and E7 may confer histone methylation and acetylation on targeted genes (Boscolo-Rizzo *et al.* 2017; Gaździcka *et al.* 2020). Histone methylation, such as elevated trimethylation at lysine 27 of histone H3 (H3K27me3), in HPV-related HNSCC is associated with tumorigenesis (Lindsay *et al.* 2017). Targeting zeste homolog 2 (EZH2), a histone methyltransferase, can reduce H3K27me3 and has the potential to sensitize cells to chemotherapy (Lindsay *et al.* 2017). Evidence also suggests that histone acetylation and deacetylation may deregulate the transcription of various genes in malignancy development in HNSCC (reviewed in (Boscolo-Rizzo *et al.* 2017; Gaździcka *et al.* 2020). Recently, Liu *et al.* demonstrated that HR-HPV oncogenes induce the long noncoding RNA (IncRNA), Inc-FANCI-2, mediated by E7 and E6, which is independent of p53/E6AP and pRb/E2F in cervical carcinogenesis (Liu *et al.* 2021). The differential regulation of lncRNAs between HPV-related and HPV-unrelated HNSCCs has also been demonstrated (Nohata *et al.* 2016; Haque *et al.* 2018; Song *et al.* 2019; Kopczyńska *et al.* 2020). However, their role in the oncogenesis of HNSCC remains elusive. In conclusion, epigenetic alterations might be useful in identifying subgroups of tumors, predicting clinical outcomes and providing potential targets (Kostareli *et al.* 2013, 2016; Ren *et al.* 2018; Shen *et al.* 2020).

In particular, research on single-cell molecular profiling can provide spatial and temporal characteristics of HNSCC in regard to intratumor heterogeneity (Puram *et al.* 2017; Qi *et al.* 2019). Tumor genetic heterogeneity determined by mutant-allele tumor heterogeneity is associated with worse outcome of patients with HNSCC (Mroz *et al.* 2013). Furthermore, a single-cell transcriptomic analysis identified subtypes as atypical, mesenchymal, basal, and classic phenotypes of OSCC (Puram *et al.* 2017). Recently, Cillo *et al.* identified a differential spectrum of immune lineages (helper CD4+ T cells and B cells) between HPV^–^ and HPV^+^ HNSCCs by single-cell transcriptional profiling (Cillo *et al.* 2020). Thus, with the advantage of single-cell technologies, a more profound understanding of genetic, epigenetic and transcriptional differences for both HPV-related and HPV-unrelated tumors would be possible (Qi *et al.* 2019).

## Impact of HPV Infection on Immune Checkpoint Blockade

**Fig. 2:**
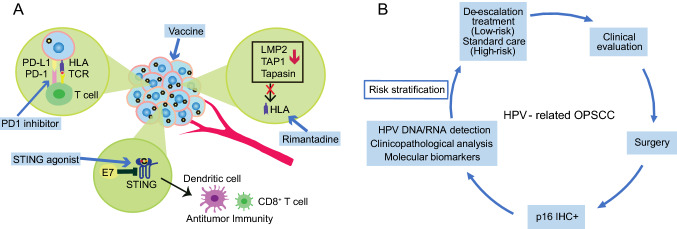
**A** Immune escape of HPV-related head and neck squamous cell carcinoma (HNSCC) and potential approaches to improve immunotherapeutic effects against HPV-related tumor. HLA: human leukocyte antigen; TCR: T-cell receptor; PD-1: programmed cell death protein 1; PDL-1: programmed death-ligand 1; LMP2: low molecular weight protein 2; TAP1: antigen processing subunit 1; STING: stimulator of interferon genes. **B** The platform of risk stratification for HPV-related HNSCC. IHC: immunohistochemical staining.

A wide exploration of the tumor-escape mechanisms of HNSCC leads to immunological approaches against tumors, including cancer vaccination and ICB treatment (Fig. 2A) (Albers *et al.* 2010; Xu *et al.* 2020a). The anti-PD-1 monoclonal antibodies nivolumab and pembrolizumab have been successfully established as first-line or second-line treatments for patients with recurrent/metastatic (R/M) HNSCC (Ferris *et al.* 2018; Cramer *et al.* 2019). The anti-PD-L1 monoclonal antibody atezolizumab has also shown its effect in patients with previously treated, advanced HNSCC in a phase I trial (Colevas *et al.* 2018). However, the overall response rates for both anti-PD1 and anti-PD-L1 treatments are only approximately 20% (Colevas *et al.* 2018; Qian *et al.* 2020). Virally mediated tumors such as HPV-related HNSCC demonstrate HPV-specific T cell immunity, and there is significant interest in developing a combination therapy to improve ICB treatment efficacy (Bhatt *et al.* 2020).

High PD-1 expression was significantly associated with HPV-positive HNSCC in an analysis of a TCGA dataset (Lyu *et al.* 2019). PDL1 expression in HPV-related HNSCC ranges from ~49.2% to ~75% according to a report (Outh-Gauer *et al.* 2020). Increased PD1 expression on T cells is correlated with HR-HPV infection in the pathogenesis of cervical intraepithelial neoplasias (CINs) (Yang *et al.* 2013, 2017). Additionally, a higher HPV 16 viral load is correlated with high CD8 + and PD-1 + TIL expression in anal squamous cell cancer (ASCC) (Balermpas *et al.* 2017). PDL1 expression is significantly enhanced in relation to HPV positivity (i.e., HPV16 E7 oncoprotein) in CINs and cervical cancer compared to normal cervical epithelia (Yang *et al.* 2013; Mezache *et al.* 2015). Taken together, these findings suggest that PD1/PDL1 pathways may be activated by HR-HPV (i.e., E5, E6 and E7 oncoproteins) for HPV-related cancers. HPV E6/E7-induced master regulators (ENO1, PRDM1, OVOL1, and MNT) are positively correlated with PD-L1 and TGFB1 expressions in cervical cancer (Qin *et al.* 2017). The activation of the PD1/PDL1 pathway by HR-HPV can further inhibit Th1 cytokine IFN-c and IL-12 expressions and upregulate TH2-type cytokine and IL-10 expressions, consequently leading to immunosuppression and CIN progression (Wakabayashi *et al.* 2019; Zhou *et al.* 2019). Remarkably, the reactivity of nonviral tumor antigen-specific T cells, including mutated neoantigen or cancer germline antigen, together with HPV oncoprotein-reactive T cells was observed in HPV-associated metastatic cervical cancer patients with complete regression after tumor-infiltrating adoptive T cell therapy (Stevanović *et al.* 2017). Thus, it would be interesting to understand the crosstalk of HPV-mediated immunosuppression and immune checkpoint activities for further development of therapeutic strategies.

Efforts to define HPV-related HNSCC responses to ICB treatments were initially assessed based on recent clinical trials (Bauml *et al.* 2017; Ferris *et al.* 2018; Burtness *et al.* 2019). In CheckMate 141, a better median overall survival among patients receiving nivolumab treatment was found to be associated with HPV-positive tumors with PDL1-positive expression (Ferris *et al.* 2018). In KEYNOTE-012, the response rate to pembrolizumab treatment varied among HNSCC patients with PD-L1 positivity, with a higher response rate in HPV-positive tumors than in HPV-negative tumors (Seiwert *et al.* 2016). A recent meta-analysis demonstrated an improved response rate to ICB treatment among patients with HPV-positive HNSCC and a higher OS in patients with PDL1-positive HNSCC (Galvis *et al.* 2020). However, the treatment efficacy of atezolizumab in a phase I trial was found independent of HPV status and PD-L1 expression level (Colevas *et al.* 2018). Because of the limited size of each study, the findings should be interpreted with caution.

The development of combination therapy by employing different immune priming approaches has the potential to improve effective immune responses in HPV-related cancer patients who receive immunotherapy. For example, targeting HPV16/18 E6/E7 by a DNA vaccine with an IL12 adjuvant is able to generate durable HPV16/18 antigen-specific cytotoxic T cells and tumor immune responses in patients with p16 + locally advanced HNSCC (Aggarwal *et al.* 2019). One patient who developed metastatic disease received anti-PD1 nivolumab treatment and had a complete response (Aggarwal *et al.* 2019). This phase I/IIa clinical trial, in line with other vaccination strategies targeted to HR-HPV E6/E7 oncoproteins, demonstrates a complementary approach to improve immunotherapy outcomes (Aggarwal *et al.* 2019; Massarelli *et al.* 2019; Xu *et al.* 2020a). The NLRX1 signaling pathway was found to be associated with HPV16 E7-mediated IFN-I suppression and TIL infiltration in HNSCC (Luo *et al.* 2020). NLRX1 depletion leads to a turnover of HPV16 E7, potentiating STING/IFN-I suppression and consequently improving tumor control (Luo *et al.* 2020). Clinical trials (NCT02675439, NCT03172936, and NCT03010176) utilizing a combination of a STING agonist plus anti-CTLA-4 or anti-PD1 ICB treatments are underway (Luo *et al.* 2020). In addition, it has been demonstrated that HPV E5 mediates resistance to anti-PD-L1 immunotherapy, which is due to acquired loss of major histocompatibility complex (MHC) expression (Sharma *et al.* 2017; Miyauchi *et al.* 2020). Rimantadine, an FDA-approved antiviral drug to treat influenza that was recently found to induce an antitumor response, could increase MHC expression in HPV E5-expressing HNSCC (Miyauchi *et al.* 2020) (Fig. 2A). However, rimantadine in combination with radiation and PD-L1 checkpoint blockade treatment did not show a synergistic effect (Miyauchi *et al.* 2020). E2-derived CD8 T cell epitopes were found in patients with HPV-related HNSCC (Krishna *et al.* 2018). Wieland et al*.* demonstrated that the E2 protein is a major target of the humoral immune response in the TME of HPV-related HNSCC (Wieland *et al.* 2020). Thus, a combination of HPV E2, E6 and E7 as targets needs to be explored for future immunotherapeutic approaches. Alternatively, innovations in nanotechnology will likely synergize with immunotherapy to elicit a robust treatment response in HNSCC (Xu *et al.* 2020a). For example, the PC7A nanovaccine, an ultra-pH -sensitive nanoparticle synergistic with anti-PD1 antibodies, can improve antitumor immunity and survival in HPV-E6/E7 TC-1 tumors (Luo *et al.* 2017). Another example is an HR-HPV nanovaccine formulated with CL 1,2-dioleoyl-3-trimethyl-ammonium-propane (DOTAP), and long HR-HPV peptides can successfully boost Ag-specific CD8 T cell responses, induce complete tumor regression through a type I IFN response in HPV-E6/E7 TC-1 tumor models and synergize with an anti-PD1 checkpoint inhibitor (Gandhapudi *et al.* 2019). It should be noted that chemoradiotherapy decreases HPV-specific T cell responses and increases PD-1 expression on CD4 + T cells in patients with HPV-related oropharyngeal cancer (Parikh *et al.* 2014). Accordingly, a future analysis of patients with HPV-related HNSCC who have and have not responded to ICB treatment would provide additional insights for targeted immunotherapy as well as deintensified treatments.

## Patient Stratification for the Deintensified Treatments

To formulate a diagnosis for HPV-related HNSCC, methods based on p16 immunohistochemical (IHC) staining, quantitative polymerase chain reaction (qPCR)-based HR-HPV DNA or RNA testing, and HPV DNA or RNA in situ hybridization (ISH) have been established. In 2018, the College of American Pathologists (CAP) and American Society of Clinical Oncology (ASCO) released guidelines that HR-HPV testing should be performed for OPSCC and cervical nodal metastases of unknown primary tumors (Fakhry *et al.* 2018; Lewis *et al.* 2018). Protein p16 IHC staining was recommended prior to other HPV testing with a cutoff of 70% nuclear and cytoplasmic positivity (Fig. 2B). For HPV-related tumors, the HR-HPV E7 oncoprotein increases expression of histone lysine demethylase 6B (KDM6B) and induces pRb degradation, thereby leading to H3K27-specific demethylation (derepression) of p16 promoter to enhance high level of p16 expression for proliferation of the cancer cells lacking the G1 checkpoint due to viral E7-induced loss of pRb (McLaughlin-Drubin *et al.* 2013; Pal and Kundu 2019). However, Albers et al*.* demonstrated that a subgroup of HNSCC with HPV-DNA^+^/p16^−^ had poor survival compared to subgroups with HPV-DNA^+^/p16^+^ and HPV-DNA^−^/p16^+^, which suggests a biologically different subtype independent of HPV status (Albers *et al.* 2017). A recent survival analysis further revealed that 60.6% of p16^+^ HPV-DNA^−^ OPSCCs did not resemble HPV16-driven but HPV-negative tumors (Wagner *et al.* 2020). Moreover, gene signatures such as TP53 mutations are indistinguishable between tumors with HPV DNA^+^RNA^−^ and those with HPV DNA^−^ (Wichmann *et al.* 2015). As indicated above, some patients who received deintensified treatments failed to show an improvement in clinical trials. Thus, pathology-based HPV testing beyond p16 IHC staining remains to be established. HPV RNA ISH, by which viral E6/E7 mRNA has been detected, showed a better survival prediction and a higher specificity than p16 IHC staining (Lewis 2020). Additionally, the sensitivity of HPV RNA ISH is higher than that of HPV DNA ISH (91% *vs*. 65%) (Kerr *et al.* 2015). The performance of HPV RNA ISH testing in stratifying OPSCC patients is worth further validation (Lewis 2020).

While current HPV-specific testing warrants further improvement, a better understanding of the biology of HPV-related HNSCC is necessary for us to identify new biomarkers correlated with disease outcomes and to predict treatment response signatures, especially for immunotherapy. For example, the latency effect of tobacco smoking exposure appears more profound in HPV-related HNSCC individuals who start smoking before sexual activity when compared to those who start after (Madathil *et al.* 2020). High levels of TILs that reflect the immune response can stratify HPV-related OPSCC patients into high-risk and low-risk groups for survival (Ward *et al.* 2014). Interestingly, a prognostic model was further developed, including three covariates (TIL levels, heavy smoking, and T-stage) in which low TIL levels, heavy smoking, and late T-stage were related to poor outcome for HPV-related OPSCC (Ward *et al.* 2014). HPV integration status based on RNA-seq data can differentiate survival between HPV integration-positive and HPV-negative HNSCC patients (Koneva *et al.* 2018). Most importantly, a set of immune-related gene signatures enriched in HPV-positive but HPV integration-negative tumors are distinguishable from those in HPV integration-positive tumors (Koneva *et al.* 2018). The lower E2F target gene expression predicted by reduced E7 levels is associated with disease recurrence through patient-derived xenograft (PDX) models for HPV-related HNSCC (Facompre *et al.* 2020). HPV + tumors have been related to an inflamed/mesenchymal phenotype. By analyzing RNA-sequencing data from TCGA, studies show that HPV^+^ HNSCC exhibited an upregulation of MHC-I- and MHC-II-related genes, which may be induced by IFN-gamma, a strong Th1 response and higher expression of the T cell “exhaustion markers” LAG3, PD1, TIGIT, and TIM3 with coordinately expressed CD39 compared to HPV^−^ tumors at the transcription level (Gameiro *et al.* 2018, 2019; Ruicci *et al.* 2019b). However, histopathologic intratumor immune cell heterogeneity is also seen within HPV-related HNSCC. Two subtypes of HPV-related HNSCC have been identified, with an inflamed/mesenchymal phenotype enriched in CD8^+^ T cell infiltration leading to better survival than a classic phenotype characterized by keratinization and higher proliferation (Keck *et al.* 2015). In addition, cancer stem cells (CSC), a subpopulation within the bulk tumor entity of HNSCC, have shown their characteristics such as less antigen expression, processing, and presentation to induce the immunogenicity and immunosuppression (Qian *et al.* 2020). Further, studies demonstrate that HNSCC patients with the HPV^+^/CSC^low^ phenotype had better outcomes than HPV^−^/CSC^high^ group (Reid *et al.* 2019). Given the paucity of molecular biomarkers, combined insights into genetic, epigenetic and transcriptional alterations can provide robust candidate biomarkers to stratify patients further and to predict treatment outcomes.

## Conclusions

The molecular landscapes of HNSCC have largely been defined during the past decade. The intratumor heterogeneity (e.g., genetic, epigenetic and histopathologic) of HPV-related HNSCC has been identified as contributing to the pathophysiology of the disease. This variability contributes to the range of treatment responses observed for both established clinical practice and clinical trials. The clinical translation of these findings may help for dynamic risk restratification of HPV-related HNSCC patients with new molecular biomarkers. The management of HPV-related HNSCC is changing, and substantial research focusing on discovery approaches to cancer diagnostics and prognostic evaluations is required (Bigelow *et al.* 2020).
